# Cortico-limbic disruption, material-specificity, and deficits in cognitive-affective theory of mind

**DOI:** 10.1093/braincomms/fcad100

**Published:** 2023-04-24

**Authors:** Varsha Singh, Kirat S Grewal, Deepti Vibha, Rajesh K Singh, Bhargavi Ramanujam, Ashima Nehra, Sarat P Chandra, Shailesh Gaikwad, Indupriya Babu, Manjari Tripathi

**Affiliations:** Psychology, Department of Humanities and Social Sciences, Indian Institute of Technology Delhi (IIT), New Delhi, 110016, India; Department of Neurology, Neurosciences Centre, All India Institute of Medical Sciences, Delhi (AIIMS), Delhi, New Delhi 110029, India; Department of Neurology, Neurosciences Centre, All India Institute of Medical Sciences, Delhi (AIIMS), Delhi, New Delhi 110029, India; Department of Neurology, Neurosciences Centre, All India Institute of Medical Sciences, Delhi (AIIMS), Delhi, New Delhi 110029, India; Department of Neurology, Neurosciences Centre, All India Institute of Medical Sciences, Delhi (AIIMS), Delhi, New Delhi 110029, India; Neuropsychology, Neurosciences Centre, All India Institute of Medical Sciences (AIIMS), Delhi, New Delhi 110029, India; Department of Neurosurgery, All India Institute of Medical Sciences (AIIMS), Delhi, New Delhi 110029, India; Department of Neuroimaging Interventional Neuroradiology, All India Institute of Medical Sciences (AIIMS), Delhi, New Delhi 110029, India; The UQIDAR Joint Ph.D. program, Indian Institute of Technology Delhi (IIT), New Delhi 110016.India; Department of Neurology, Neurosciences Centre, All India Institute of Medical Sciences, Delhi (AIIMS), Delhi, New Delhi 110029, India

**Keywords:** cognitive-affective integration, material-specificity, right medial temporal epilepsy, theory of mind

## Abstract

The Theory of Mind deficit due to cognitive-affective disintegration is a poorly understood cognitive consequence of cortical and subcortical disruption in right temporal lobe epilepsy. Following Marr's trilevel approach, we used the material-specific processing model to understand the Theory of Mind deficit in drug-resistant epilepsy (*N* = 30). We examined pre- and post-surgery changes in first-order (somatic-affective, non-verbal component) and second-order Theory of Mind (cognitive-verbal component) in three groups formed using: (i) seizure side (right versus left), (ii) right temporal epilepsy (right temporal lobe epilepsy versus non-right temporal lobe epilepsy), and (iii) right temporal lobe epilepsy with amygdalohippocampectomy (right temporal lobe epilepsy versus left temporal lobe epilepsy amygdalohippocampectomy versus non-amygdalohippocampectomy). We observed a marked deficit in the first-order Theory of Mind in the right temporal lobe amygdalohippocampectomy group; we mapped this deficit to decline in the non-verbal component of Theory of Mind (somatic-affective component). Preliminary results support using a material-specific processing model to understand the Theory of Mind deficits in right temporal lobe epilepsy amygdalohippocampectomy. Malleability of verbal processing in presence of deterioration of non-verbal processing might have clinical relevance for post-surgery recovery in right temporal lobe epilepsy amygdalohippocampectomy. Documenting the material-specific nature of deficits (verbal versus non-verbal) in non-western, linguistically, and socioeconomically diverse country enables us to understand the problem of heterogeneity in post-surgery cognitive consequences in the right amygdalohippocampectomy.

## Teaser

Post-surgery cognitive deficits in temporal lobe epilepsy are poorly understood; Singh *et al*. applied material-specific processing model to Theory of mind deficit and observed that cognitive-affective disintegration in right temporal epilepsy with cortico-limbic disruption (amygdalohippocampectomy) showed first order Theory of mind deficit, potentially due to material-specific alteration of non-verbal processing.

## Graphical Abstract

**Graphical abstract:**
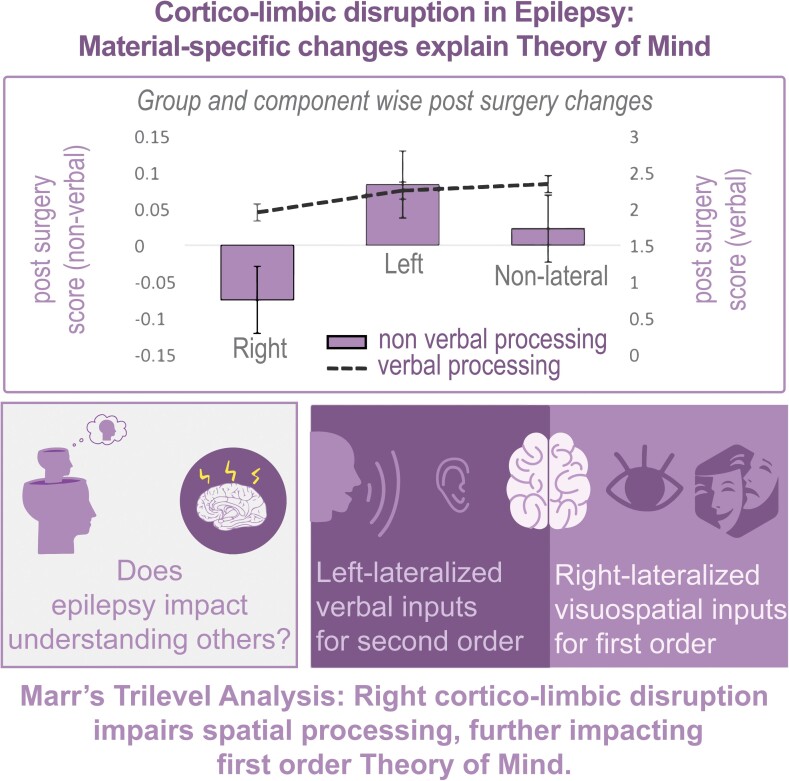

## Introduction

Temporal lobe epilepsy (TLE) is the most common drug-resistant epilepsy, and the treatment involves brain resection surgery for seizure-cessation. Although epilepsy is a network disorder involving bilateral epileptogenic networks, TLE shows a lateralized seizure origin with subcortical involvement of the hippocampus, amygdala, or entorhinal cortex.^1^ Over the years, selective unilateral resection of amygdalohippocampectomy (AH) has been preferred over temporal lobe resection due to seizure-control benefits that minimize disruption of cognitive functions. The hippocampus and amygdala within the medial temporal cortex are crucial for multimodal information integration. Specifically, integrating information from the external world with the consequent body states (i.e. interoception) enables successful interaction with the world while maintaining internal homeostasis balance.^2^ Documenting the structural and the functional consequences of AH in unilateral TLE aids our understanding cognitive-affective disintegration in TLE, which is critical for minimizing cognitive deficits in individuals with TLE. The choice of surgery depends on the extent of presurgery functional specialization, commonly determined by language lateralization (e.g. left side language dominance).

Further, postoperative deficits show laterality that abides by the material-specificity principle, that is, verbal processing deficits are associated with the left side AH, whereas spatial/perceptual deficits are observed in the case of right side surgery.^3,4^ Although post-surgery changes that engage cognitive processes and are anatomically distinct, forms the crux of post-surgical assessment in TLE, this traditional binary approach of ‘deficit–no deficit’ camouflages alteration of distinct, delineated cognitive processes.^5^ For instance, uncertainties of social interactions that rely on cognitive and affective processing could be linked with alterations due to AH resection and cognitive-affective disintegration. The right side AH shows a greater deficit in emotion processing, whereas more significant cognitive/verbal impairment is observed in the left AH.^6,7^ The perplexing phenomenon of social deficit, despite intact cognitive/verbal processing in the right AH, may be due to disruption in non-verbal interoception cues.^8^ The right TLE participants show poor ability to detect others’ emotions compared to the non-TLE (extra TLE) or left TLE participants.^9^ It is clinically relevant to understand the reasons for greater severity of social deficit in right TLE participants compared to participants with non-TLE or left TLE.^10^ We suspect the medial temporal lobe connectivity to subcortical, limbic neural circuitry, specifically higher amygdala activation in the right TLE, contributes to the social deficit in the right TLE.^11^ In other words, right TLE and cortico-limbic disruption might hamper cognitive and affective integration required for social cognition. We use Marr’s^12^ tri-level framework (1982) to examine social deficit in right TLE, explicitly aiming to understand postoperative cognitive and structural changes at computational (what), algorithmic (how), and implementation level (where).

The Theory of mind (ToM) posits that the ability to mentalize others’ thoughts is critical for carrying out social interactions; mentalizing involves cognitive (cognitive inference of others’ thoughts/beliefs) and somatic-affective components (somatic-affective inference of others’ emotional states).^13,14^ We use the material-specific processing model to explain ToM deficit in the right TLE. Our conjecture is as follows: somatic-affective experiences of good and bad outcomes help us interpret the external cues available to our sensory system (e.g. facial expressions, actions), and such somatic-affective cues also help us predict others’ thoughts and actions. To illustrate, we present an example: a patient sees the nurse put the medicine in a box and leave the room; if someone removes the medicine from the box, the nurse is likely to think that the medicine will still be in the box when she returns. In this case, the patient's sensory information differs from that of the nurse (i.e. visual input of the medicine’s removal is available to the patient, and under normal conditions, the patient is aware that the visual input is not available to the nurse). Mentalizing ability involves being aware of this difference in the availability of sensory information; the patient’s thoughts/belief about the object’s location (medicine) differs from those of the nurse, and the patient is aware that the nurse holds a false belief about the object's location. The patient’s belief about others enables them to predict thoughts (e.g. the nurse's false belief about the object's location) and actions of others (e.g. the nurse's action when accessing the object).

Mentalizing others’ thoughts and predicting their actions based on sensory information helps reduce social uncertainties and facilitates social interactions. However, it is unclear how far our sensory, somatic-affective feedback can go while predicting others’ thoughts and actions. Not all somatic-affective states and thoughts of others are fully or directly available to our sensory system; some objects, people, or events might be connected to us remotely or indirectly via another object, person, or event. Without first-hand sensory and somatic-affective information, our somatic-affective states could be insufficient for predicting others’ beliefs. In the ToM framework, first-order predictions rely on observable cues of somatic-affective information (cues that are available to our observation) and help predict others’ thoughts (e.g. ‘she sounds passionate about free speech, she might think that the rally is important’). These are distinct from the second-order prediction of ToM, predicting others’ thoughts about others (e.g. ‘she thinks he is passionate about free speech and he will think that the rally is important’). As the distance between the perceiver and the others increases (as in the case of second-order ToM), the somatic-affective cues become more insufficient. In other words, our somatic-affective cues (facial expressions, language tone, and actions) about the other person's mental representation of others (i.e. what she/he interacts, reacts, and think of others) are limited compared to the somatic-affective cues that form our mental representation of the other person (i.e. what s/he interacts, reacts, think towards us). The first-order prediction might reflect dominant cognitive-affective integration.

Due to the insufficiency of somatic-affective information from the observable cues (actions, face, and tone), the second-order prediction might rely on cognitively demanding processes such as context-related reasoning, logic-abstraction, and linguistic inferences. This postulation of second-order belief and linguistic reliance aligns with the development studies that indicate that first-order ToM develops before the second-order ToM, and language is crucial for second-order ToM.^15^ Therefore, the material-specific processing model suggests that the first-order ToM prediction predominantly relies on somatic-affective cues (non-verbal), and the second-order prediction predominantly relies on cognitive, linguistic inference (verbal). We assume that ToM (first versus second-order prediction) and material-specific processing model (verbal versus non-verbal components) will help us understand how cognitive-affective disintegration and alteration account for postoperative ToM deficits in the right TLE.

Assessing postoperative functional and structural changes due to cortico-limbic alterations is critical but challenging in a network disorder such as epilepsy.^16^ Because of higher parental other-referencing prevalent in non-western cultures, we assume long-term reliance on mentalizing as a cognitive function, and therefore, we expect that there might be prominent structural and functional alteration in ToM mentalizing.^17,18^ We examine pre- and post-surgery alteration in ToM to understand the changes in the functional capacity (mentalizing: first versus second-order ToM), the sub-processes contributing to it (material-specific processing: somatic-affective, non-verbal versus cognitive, verbal processing), under cortical (lateralization: functional and lobar) and subcortical disruption (surgical resection: amygdala–hippocampus versus none) might influence cognitive function of mentalizing others thoughts. According to the material-specific processing model, we expected that cognitive-affective disintegration associated with cortico-limbic circuitry in the right TLE would account for the ToM deficit.

## Materials and methods

### Sample

A total of 30 drug-refractory epilepsy patients (mean age: 24.8 ± 10.54 years; male = 21) admitted to the neurology ward (from January 2018 to December 2018) were screened for inclusion criteria and followed for four months after epilepsy surgery (the term ‘patient’ is used for methodological detail and accuracy). We followed the procedure adopted by others, where participants who met an inclusion criterion and consented to participate served as a sample (see Table 1).^11,19–21^ The mean age of onset of seizures was 11.2 ± 10.09 years, with a mean duration of 13.5 ± 8.7 years. The type of seizure was focal, with impaired awareness in all patients. The mean monthly seizure frequency was 16 ± 14.9, and the median number of antiepileptic drugs was three (2–5). Out of these patients, a total of 22 patients were of TLE (with ten patients of dominant and 12 patients of non-dominant TLE) and eight patients of extra TLE. Long-term video EEG localization was right temporal in 13 patients (43%), left temporal in nine patients, right frontal in four patients, left frontal in three patients, and right parietal in one patient. Epilepsy surgery performed was anterior temporal lobectomy with AH in 19 patients (63%), electrocorticography-guided resection in nine patients (30%: out of which five were frontal, three temporal, and one parietal), and hemispherectomy in two patients. Participants were classified into comparison groups by combining seizure localization from neurological features related to epilepsy (video EEG, nature of resection) as depicted in Table 2.

**Table 1:** Demographics and clinical characteristics

	Right temporal AH (*N* = 10)	Left temporal AH (*N* = 9)	Non AH (*N* = 11)
Age (years) Mean ± SD	30 (±11.5)	25.2 (±11.36)	19.6 (±7.06)
Sex M/F	7/3	6/3	8/3
Right Handedness	9	7	8
Age at onset(years)Mean ± SD	14.6 (±12.58)	10.6 (±10.95)	8.6 (±6.16)
Febrile convulsion	10%	0%	0%
Frequency Mean ± SD	8.9 (±12.2)	12 (±10.72)	25.45 (±16.17)
Duration Mean ± SD	15.5 (±11.94)	14.5 (±6.08)	10.90 (±7.13)

Note: AH = Amygdalohippocampectomy, SD = Standard Deviation; M = Male, F = Female; Total *N* = 30.

**Table 2:** Group comparison of pre-post-operative changes in Theory of mind (ToM)

Epilepsy-related feature	Group comparison (*n*)
Seizure localization: side	R side (18) versus L side (12)
Seizure localization: temporal lobe (TL)	RTL (13) versus non-RTL (17)
AH resection: temporal lobe (TL AH)	RTL AH (10) versus L TL AH (9) versus non-AH (11)

Note: AH: Amygdalohippocampectomy; R = Right, L = Left; TL: Temporal lobe.

### Material

ToM assessment: Social Cognition Rating Tools in Indian Setting,^22^ a culturally-adapted evaluation of the ToM was undertaken (Refer to Supplementary material). The assessment included four short stories and two stories on metaphor and irony; each was used to calculate the first-order ToM and second-order ToM (in the form of an index (composite scores reflecting non-verbal belief processing and verbal and linguistic processing). The first-order ToM index consists of two short stories and two stories on metaphor, and the second-order ToM index consists of two short stories and two stories on irony. Metaphors are processed as a figurative aspect of a language and rely on the first-order ToM. In contrast, irony depends on the linguistic aspect of language and is linked with the second-order ToM.^23^ First-order ToM index was expected to reflect the dominance of somatic-affective, non-verbal components, and the second-order ToM index was expected to reflect the dominance of cognitive, verbal components. The conventional assessment includes ten stories on faux pas that gives a Faux Pas composite index (FPCI), and eight audiovisual clips in the vernacular language give a social perception index (SPI).

### Variables and statistical analysis

The variables derived from the ToM assessment, namely, the first-order ToM index and second-order ToM index, were ordinal variables. Pre- and post-surgery scores were used to analyze pre- and post-surgery changes in the ToM, and the difference in post- minus presurgery scores were used for component-wise analysis and material-specificity. These scores served as within-subject variables for mixed analysis of variance (ANOVA), with the group as a between-subject variable. The first grouping of patients was done based on seizure localization on the right (18) versus left side (12), the second involving the right temporal lobe (13) versus non-right temporal lobe (i.e. nine left temporals, three left frontals, four right frontals, one right parietal, total = 17), and lastly, grouping based on the temporal lobe with AH, that is patients with right temporal AH (10), left temporal AH (9), non-AH (11). Scores of first-order ToM and second-order ToM were analyzed separately. Each component that forms the ToM index (non-verbal and verbal components) was analyzed individually and then combined in a material-specific and non-specific way. Non-parametric substitutes of ANOVA were used (Friedman's test with follow-up Wilcoxon signed-rank test) to address concerns related to the small sample size in sub-groups. We addressed concerns related to the heterogeneity of the small sample by repeating the analyses for each variable that could potentially impact material-specific pots-surgery outcomes, namely, sex, age, intelligence, education, epilepsy duration, and seizure frequency. Data-split was for sex, and cut-offs that divided the sample based on age, intelligence, education level, epilepsy duration, and seizure frequency allowed us to examine material-specific hypotheses in post-surgery differences in non-verbal and verbal processing attributed to cortico-limbic disruption varies across these critical factors.

### Procedure

The longitudinal study was carried out at a national center; patients were screened based on the inclusion criteria. Patients with drug-refractory epilepsy with age ≥5 years admitted for epilepsy surgery under Neurology unit III, Department of Neurology from January 2018 to December 2018 participated in the study. All patients or legal representatives provided written informed consent according to the Declaration of Helsinki, and its amendments.^24^ The institute ethics committee of AIIMS approved the protocol [Ref.No.IECPG-470/29.11.2017]. Drug refractory epilepsy was defined as the failure of adequate trials of two or more tolerated, appropriately chosen, and appropriately used antiepileptic drug regimens. Baseline characteristics of the study population, including age, sex, birth and milestone history, onset and duration of epilepsy, seizure frequency, and the number of antiepileptic medications, were collected. Patients with severe mental retardation were excluded after consulting the neuropsychology assessment. Preoperative assessments of all patients were documented as per protocol. These included neurological history/examination, long-term video-EEG monitoring, MRI Brain, SPECT, PET Brain, and invasive video-EEG when necessary. We made attempts to reduce study biases in the following ways: (a) using of culturally-suitable measure of ToM reduced selection bias as linguistic barriers did not limit participation, (b) collecting detailed records for detailed patient information (c) in the absence of healthy control group, pre- and post-surgery analyses were carried out on participants using three classifications/groupings, (d) maintaining a uniform gap of 4 months to carrying out ToM assessment with post-surgery neurological follow-up enabled zero attrition at follow-up. Standard Neuropsychological testing, including assessment of handedness, was carried out for all patients. Resective epilepsy surgery was performed after informed consent and per eligibility and consensus of the epilepsy surgery board. We administered Social Cognition Rating Tools in an Indian Setting to the eligible patients before surgery and at least four months post-surgery.

### Data availability

The data supporting this study's findings are available from the corresponding author upon reasonable request.

## Results

The baseline demographic and clinical characteristics of the sample are listed in Table 1. The ToM scores served as variables and were analyzed to examine the effect of right side seizure (right side versus left side), right temporal lobe as seizure localization (R TL versus non-R TL), and AH surgery (R TL versus L TL versus none). The seizure localization groups were as follows: surgery/seizure side group (right side versus left side), right temporal lobe seizure localization group (right temporal lobe versus non-right temporal lobe), and right temporal AH group (right temporal lobe AH versus left temporal lobe AH versus non-AH group) as listed in Table 2.

Our plan for analyses: (1) in the conventional ToM assessment, scores of two components (first-order belief and metaphor) are combined to form a first-order ToM index (somatic-affective dominant), and second-order belief and irony scores form the second-order ToM index (cognitive dominant). We delineated these composite scores to differentiate between beliefs (first & second order) as a non-verbal component distinct from language (metaphor & irony) as a verbal component of ToM assessment. We analyzed changes in the components of the ToM assessment to reflect somatic-affective versus cognitive demands within the material-specific processing model (non-verbal belief processing versus verbal processing). (2) Post and presurgery differences were calculated to reflect post-surgery improvement (post- minus presurgery scores) for each of the four components of ToM scores: first-order belief scores, second-order belief scores, metaphor processing scores, and irony processing scores. A positive value reflected the post-surgery improvement in scores, whereas negative values indicated a decline in scores. (3) We examined the groups formed due to the right seizure side, groups involving the right temporal lobe, and groups of the right temporal lobe with AH. This approach enabled us to examine the extent to which right-lateralized cortico-limbic disruption influences changes in the somatic-affective component versus the cognitive component of ToM (first versus second-order beliefs), the cognitive component of verbal-linguistic/language processing of ToM (metaphor versus irony), the material non-specific model tested somatic-affective component of ToM (first order belief and metaphor versus second-order belief and irony), and lastly material specific model tested non-verbal versus verbal component (first and second order belief versus metaphor and irony).

We expected that mentalizing about others in the ToM assessment is either driven by the somatic demands of first-order processing versus cognitive demands of second-order processing or, according to the material-specific processing model, somatic demands of belief processing versus the cognitive demands of verbal processing. The results address: (a) ToM alteration: changes in the conventional first and second-order ToM index examined separately to understand the seizure and surgery impact on cognitive-affective integration requisite for ToM. We deducted post-surgery scores from the presurgery scores to reflect potential improvement (as a positive score) or deficit (as a negative scores), it allowed us to delineate and visualize material specific alterations in composite and component-wise scores (b) Component-wise alteration: Analysis of the beliefs/non-verbal component of ToM (first-order belief versus second-order belief) and the verbal components of ToM (metaphor versus irony processing) (c) Material-specific and non-specific alteration: changes were analyzed to examine alteration in material non-specific composite score (first order belief + metaphor processing versus second-order belief + irony processing), and in material-specific composite score (first order belief + second order belief versus metaphor processing + irony processing) (d) Heterogeneity in post-surgery alteration: material-specific composite score (first order belief + second order belief versus metaphor processing + irony processing) was further examined against factors that could impact material-specific processing of verbal and non-verbal components of ToM (i.e. age, sex, intelligence, education, epilepsy duration, and seizure frequency).

### A. Theory of mind

First order (somatic-affective) and second-order ToM (cognitive) The effect of seizure side on first order ToM index showed no main effect *F* (1, 28) = 0.73 *P* = 0.40, the two-way interaction of seizure side and first order ToM index was not significant *F* (1, 28) = 0.73 *P* = 0.40. The effect of seizure side on second order ToM index showed no main effect *F* (1, 28) = 0.94 *P* = 0.34, the two-way interaction of seizure side and first order ToM index was not significant *F* (1, 28) = 1.61 *P* = 0.21. The effect of seizure side were examined on other subscale scores as well, FPCI showed no main effect *F* (1, 28) = 0.07 *P* = 0.80, two-way interaction of seizure side and FPCI was not significant *F* (1, 28) = 0.06 *P* = 0.81. For SPI, the main effect was not significant F (1, 28) = 0.11 *P* = 0.74, and the two-way interaction was not significant *F* (1, 28) = 0.86 *P* = 0.36. For SPI-abstract (SPI-A), the main effect was not significant *F* (1, 28) = 1.03 *P* = 0.32, and the two-way interaction was not significant *F* (1, 28) = 0.20 *P* = 0.66.

The effect of seizure localization in the right side temporal lobe was examined, first order ToM showed no main effect *F* (1, 28) = 0.37 *P* = 0.55, and the two-way interaction of seizure side and first order ToM scores was not significant *F* (1, 28) = 0.37 *P* = 0.55. There was no main effect on second order ToM *F* (1, 28) = 0.34 *P* = 0.56, the two-way interaction of seizure side and first order ToM scores was not significant *F* (1, 28) = 0.10 *P* = 0.33. The effect on FPCI was not significant *F* (1, 28) = 0.01 *P* = 0.93, the two-way interaction of seizure side and FPCI was not significant *F* (1, 28) = 0.86 *P* = 0.36. For the SPI, the main effect was not significant F (1, 28) = 0.47 *P* = 0.50, and the two-way interaction was not significant *F (*1, 28) = 1.34 *P* = 0.26. For SPI-A, the main effect was not significant *F* (1, 28) = 1.79 *P* = 0.19, and the two-way interaction was not significant F (1, 28) = 1.93 *P* = 0.18.

**Figure 1:**
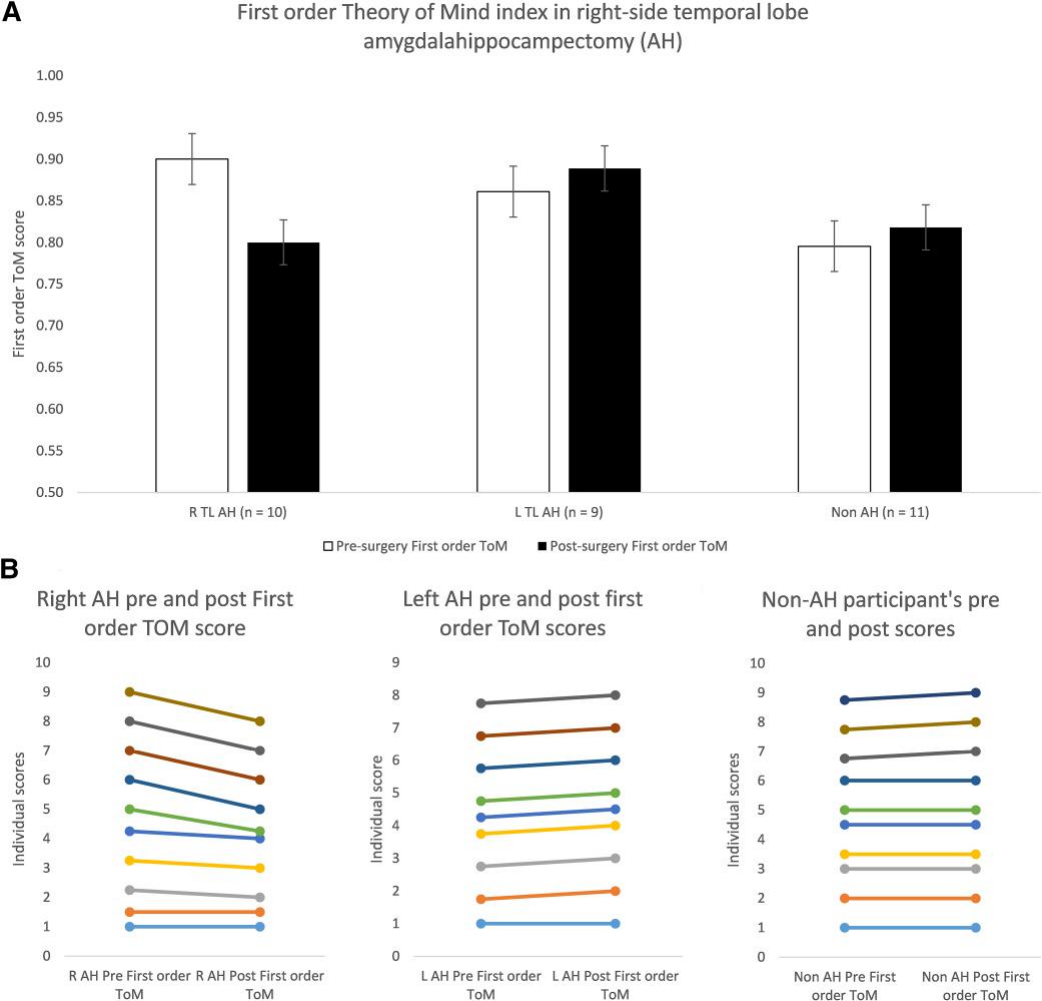
**(A) Group-wise scores of first order theory of mind index.** First-order theory of mind (ToM), R: Right, L: Left, TL: Temporal lobe, AH: Amygdalohippocampectomy, Error bars represent standard error. The analysis of variance showed significant two-way interaction of group and first-order ToM scores, *F* (2, 27) = 3.63 *P* = 0.04. In contrast to the left AH and non-AH groups, right temporal AH group showed a slight decline in first-order ToM index score (mean 1 = 0.90, mean 2 = 0.80). **(B)** Individual pre−post-surgery scores of first order Theory of Mind (group-wise). Individual scores of pre- and post-surgery First order Theory of Mind index presented group-wise. In the non-AH group (*n* = 11), one participant had a pre- and post-score of zero (i.e. one participant scored ‘0’ for pre- and post-surgery assessment, therefore data/line for that participant does not appear in the graph depicting non-AH group’s individual scores).

We examined the effects of surgery for participants with right temporal localization with AH, those with left temporal localization with AH, and participants without AH. The first order ToM showed no main effect *F* (1, 27) = 0.56 *P* = 0.46, the two-way interaction of group and first-order ToM scores was significant *F* (2, 27) = 3.63 *P* = 0.04 partial eta squared = 0.21. The right temporal AH group showed a decline in first-order ToM index (mean 1 = 0.90, mean 2 = 0.80), whereas the left temporal AH (mean 1 = 0.86, mean 2 = 0.89), and the non-AH surgery group showed slightly improved first-order ToM scores (mean 1 = 0.79, mean 2 = 0.82). Mauchy's and Levene's tests showed no concern. The effect on second order ToM was not significant *F* (1, 27) = 0.67 *P* = 0.42, the two-way interaction of group and first-order ToM scores was not significant *F* (2, 27) = 1.38 *P* = 0.27. The effect on FPCI was not significant *F* (1, 27) = 0.02 *P* = 0.89, the two-way interaction of group and FPCI was not significant *F* (2, 27) = 1.37 *P* = 0.27. For SPI, the main effect was not significant F (1, 27) = 0.21 *P* = 0.65, the two-way interaction was not significant *F* (2, 27) = 0.42 *P* = 0.66. For SPI-A, the main effect was not significant F (1, 27) = 1.14 *P* = 0.29, the two-way interaction was not significant F (2, 27) = 0.15 *P* = 0.86 (see Fig. 1).

### B. Component-wise alteration: Non-verbal (belief) and verbal processing

Changes in the conventional assessment of the ToM index comprise scores of first-order ToM (first-order belief & metaphor: somatic-affective) versus second-order ToM (second-order belief & irony: verbal-linguistic). This assessment combines non-verbal and verbal components and is treated as a material non-specific model (i.e. the alteration reflects beliefs (non-verbal) and verbal processing). Delineating non-verbal belief processing from verbal processing offers insights into the material-specific alteration of right-lateralized cortico-limbic disruption. Therefore, scores of belief processing were separated from verbal processing to carry out component-wise analyses of post-surgery change (i.e. post-surgery score minus presurgery score where positive value reflects post-surgery increase).

**Figure 2:**
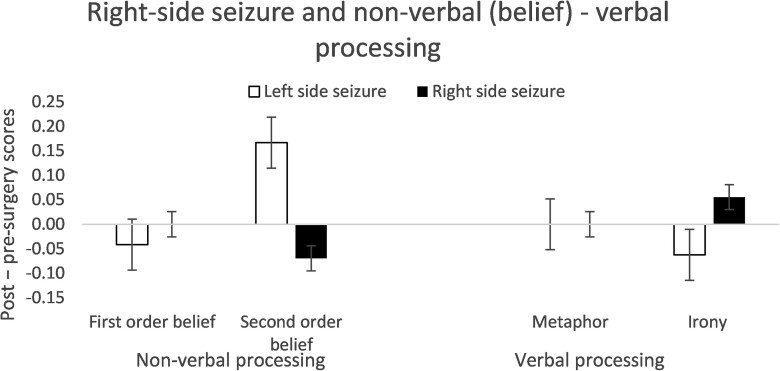
**Right-side seizure: non-verbal (beliefs) and verbal component.** The results of analysis of variance indicated that the two-way interaction of right versus left side seizure localization group and component-wise post-surgery alteration, group × belief was significant *F* (1, 28) = 4.67 *P* = 0.039 (left side bars in the figure show results for belief). The two-way interaction of right-side seizure group × verbal processing component was non-significant (right side bars in the figure show results for verbal processing).

We examined alteration in the non-verbal component, that is, belief processing in first-order (somatic-affective) versus second-order false beliefs (cognitive). For the right side seizure localization group, the main effect was non-significant (*P* = 0.289) indicating no difference in the two types of beliefs, the two-way interaction post-surgery difference in beliefs × group was significant *F* (1, 28) = 4.67 *P* = 0.039 partial eta squared = 0.14, the left side seizure localization showed a decline in first-order belief and improvement in the second-order belief (mean for first order belief = −0.042 and mean for second order belief = 0.167). In contrast, the right side seizure localization showed stable first-order belief processing (mean for first-order belief = 0.0), second-order belief scores declined (mean for second-order belief = −0.069) (Fig. 2 shows the interaction of non-verbal processing of first and second-order beliefs for the right-side versus left-side seizure group). The result for the right temporal group showed no main effect (*P* = 0.697), and the two-way interaction failed to meet the statistical significance (*P* = 0.070). The results for right temporal localization with AH showed no main effect (*P* = 0.532), and the two-way interaction was non-significant (*P* = 0.662).

**Figure 3:**
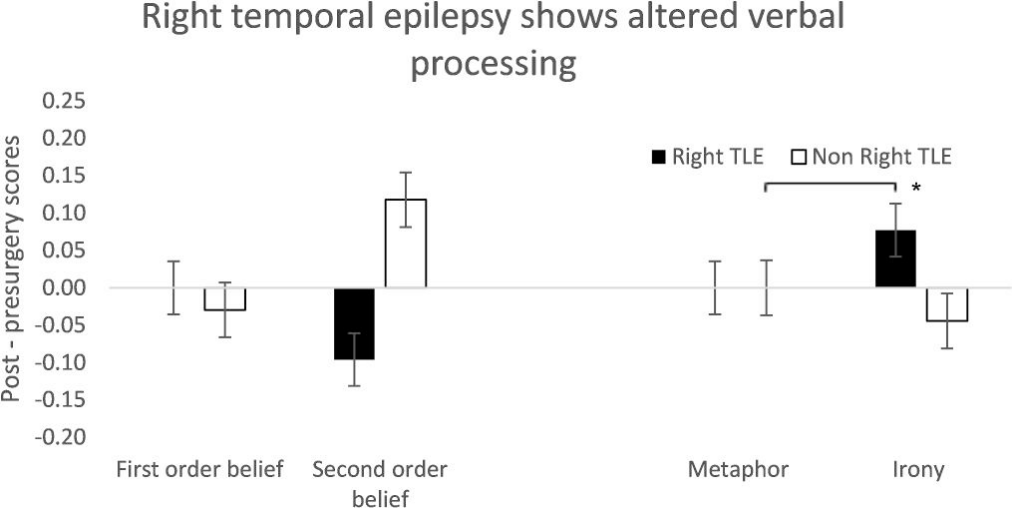
**Right temporal epilepsy (Right TLE): non-verbal (belief) and verbal component**. The results of analysis of variance indicated that the two-way interaction of right temporal lobe epilepsy (right TLE) versus non-right TLE group and component-wise analysis, TLE group × verbal component (metaphor versus irony) was marginally significant *F* (1, 28) = 4.302 *P* = 0.04, right TLE group showed slight improvement in irony processing (right side bars in the figure show these results). The right TLE group × belief component (first-order belief versus second-order belief) was non-significant (left side bars show these results).

Change within the verbal component was examined by comparing metaphor (somatic-affective) versus irony processing (cognitive). For the right side seizure localization group, the main effect was non-significant *P* = 0.908, indicating no difference in verbal processing components of metaphor and irony, the two-way interaction of group × verbal processing failed to meet statistical significance *F* (1, 28) = 3.957 *P* = 0.057. The results for the right temporal lobe seizure localization showed non-significant main effect for changes in metaphor versus irony (*P* = 0.578), the two-way interaction was significant *F* (1, 28) = 4.302 *P* = 0.047 partial eta squared = 0.133, the right TLE group showed stable/unchanged metaphor processing but a slight improvement in irony processing was observed (metaphor processing mean 1 = 00, irony processing mean 2 = 0.077) whereas the non-right temporal lobe seizure group showed a small decline in irony processing compared to stable/unaltered metaphor processing (metaphor processing mean 1 = 000, irony processing mean 2 = −0.044). The results for right temporal localization with AH showed no significant main effect (*P* = 0.726) or interaction effect (*P* = 0.110). Levene’s test showed no concern for equality of error variance for the group analyses (*P* > 0.05) (see Fig. 3).

### C. Material specificity in post-surgery alterations

We examined the non-material-specific changes in the ToM composite index (combined scores), the total of the first-order belief and metaphor processing versus the total of second-order belief and irony. The right side seizure group showed no main effect of first-order versus second-order ToM alterations (*P* = 0.208), and the two-way interaction of ToM index alteration and groups was non-significant (*P* = 0.130). The right temporal lobe seizure localization group showed no main effect of ToM alterations (*P* = 0.423), and the two-way interaction with the group was non-significant (*P* = 0.246). The results for right temporal localization with AH showed no significant main effect (*P* = 0.318) or interaction effect (*P* = 0.695).

Next, material-specific alterations in material-specific combined scores, that is, non-verbal belief processing (first and second-order beliefs: somatic-affective) versus verbal processing (metaphor & irony: verbal-linguistic), were examined. For the right side localization, main effect was highly significant *F* (1, 28) = 116.26 *P* = 0.000, partial eta squared = 0.806, non-verbal belief processing showed slight increase compared to the robust improvement observed in verbal processing (mean 1 = 0.028 and mean 2 = 2.142). The two-way interaction was non-significant (*P* = 0.135), indicating that the right-side seizure localization did not contribute to the difference in the post-surgery composite improvement of non-verbal versus verbal processing. For the right TLE, the main effect was significant *F* (1, 28) = 128.04 *P* = 0.000, partial eta squared = 0.821, non-verbal, belief processing deteriorated whereas the verbal processing showed an improvement (mean 1 = −0.004 and mean 2 = 2.207). The two-way interaction was non-significant (*P* = 0.178), indicating that the difference in non-verbal and verbal processing was independent of right TLE. The results for the right temporal localization with AH group showed a significant main effect *F* (1, 28) = 114.14 *P* = 0.000 partial eta squared = 0.810. The two-way interaction effect was non-significant (*P* = 0.833), indicating that the participant grouping (right AH, left AH, non-AH) did not contribute to the difference in processing. We observed that the prominent changes supported the material-specific processing model (e.g. improved verbal processing compared to non-verbal belief processing).

Despite the distinct decline in first-order ToM in right AH (see Fig. 1), the component-wise alterations (belief & verbal processing), the material non-specific and the material-specific processing models showed no effect of cortico-limbic disruption of right temporal lobe with AH. We used a non-parametric substitute of repeated measures to examine post-surgery alterations in AH groups (right, left, & non-AH) to rule out the small sample size of participant subgroups in the AH grouping (i.e. the left AH group had less than 10 participants). We repeated the component-wise analysis [i.e. belief processing (first versus second-order belief) & language processing (metaphor versus irony)], the material non-specific (first-order belief + metaphor versus second-order belief + irony) and the material-specific analysis (first + second order belief versus metaphor + irony) using data-split for the three AH groups (right AH, left AH, and non-AH). Friedman's test allowed us to examine paired differences in the components for non-verbal, verbal, material non-specific, and material-specific processing. The results indicated that the three groups showed changes aligned with the material-specific processing model (see Table 3); post-surgery alteration in the three AH groups differed significantly only for paired comparison of beliefs (non-verbal) versus language (verbal) components of ToM.

**Table 3:** Group-wise pre- and post-surgery difference for the paired/repeated components

Model	Components/repeated measure (difference in post–presurgery scores)	Surgery Groups	Chi-square	df	*P* value
Component-wise	Non-verbal/belief (first versus second order belief)	Right AH (10)	0.667	1	0.414
		Left AH (9)	0.000	1	1.000
		Non AH (11)	0.111	1	0.739
Component-wise	Verbal/Language (metaphor versus irony)	Right AH (10)	2.000	1	0.157
		Left AH (9)	3.000	1	0.083
		Non AH (11)	2.000	1	0.655
Material non-specific (conventional)	Theory of mind index scores (first order belief + metaphor) versus (second order belief + irony)	Right AH (10)	0.667	1	0.414
		Left AH (9)	3.000	1	0.083
		Non AH (11)	0.667	1	0.414
Material-specific	Non-verbal component of Beliefs (first order + second order belief) versus verbal component of language (metaphor + irony)	Right AH (10)	10.00	1	0.002
		Left AH (9)	9.000	1	0.003
		Non—AH (11)	10.000	1	0.002

**Figure 4:**
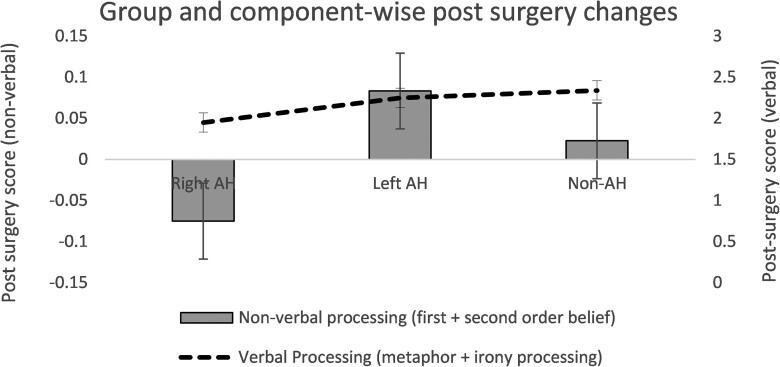
**Group-wise post-surgery changes in non-verbal (belief) & verbal components.** Non parametric group-wise comparison (Wilcoxon signed rank) indicates right AH ToM deficit in the non-verbal component (first and second order false belief processing).

*Post hoc* analysis in Wilcoxon signed-rank test with data-split for AH groups (right AH, left AH, & non-AH) indicated that in the right AH group, post-surgery change in the individual components, the non-verbal processing (first versus second-order belief processing: *P* = 0.339) and the verbal processing (metaphor versus irony processing: *P* = 0.180), and the material non-specific processing (first order + metaphor versus second order + irony: *P* = 0.750) was non-significant. Only the material-specific alteration showed significant alteration (first + second order belief versus metaphor + irony: *Z* = -2.812, *P* = 0.005), such that the right AH group showed deterioration of non-verbal (belief) processing (mean = −0.075) whereas the verbal processing showed an improvement (mean = 1.95). For the left AH group, the post-surgery alteration of non-verbal processing (first versus second-order belief: *P* = 0.739), verbal processing (metaphor versus irony: *P* = 0.083), and material non-specific processing (first-order + metaphor versus second-order + irony: *P* = 0.102) was non-significant; only material-specific alteration was significant (first + second order belief versus metaphor + irony: Z = -2.670, *P* = 0.008), the left AH group showed marginal improvement in non-verbal processing (belief) (mean =0.0833) compared to verbal processing (mean = 2.250). For the non-AH group, the difference in post-surgery improvement for non-verbal (first versus second-order belief: *P* = 0.903), verbal (metaphor versus irony: *P* = 0.655), and material non-specific processing was non-significant (first order + metaphor versus second order + irony: *P* = 0.914). Only material-specific alteration showed significance (first + second order belief versus metaphor + irony: Z = -2.809, *P* = 0.005), the non-AH group showed a marginal improvement in non-verbal (belief) (mean = 0.0227) compared to verbal processing (mean = 2.340). Although preliminary, these results indicate a possibility of post-surgery decline in the non-verbal component of belief as a unique feature of the right AH group (see Fig. 4).

### D. Heterogeneity in material-specific alteration

It is difficult to match participants on numerous factors that influence somatic and cognitive processing of participant subgroups contributing to heterogeneity in post-surgery outcomes. For instance, the material-specific processing model accounted for post-surgery changes in the right AH group, where verbal and non-verbal components of ToM showed alteration. However, these material-specific alterations might differ due to age, sex, handedness, intelligence, education level, duration of epilepsy (years), and seizure frequency. Therefore, we repeated the material-specific analysis of non-verbal component of belief (first order + second order belief) versus verbal component of language (metaphor + irony) in the three AH groups (right AH versus left AH versus non-AH) with data-split by variables of interest: age (≤22 = 50%), sex (male = 70%), handedness (right-handed = 80%), intelligence (low = 40%), education (≤8th grade) = 50%), duration of epilepsy (≤10.5 years = 50%), seizure frequency (≤8 = 53.3%). The post-surgery outcomes of the right AH was accounted by material-specific processing of ToM applicable in case of right-handed male adults (≥22 years) with mid-level intelligence and school education above middle school, experiencing ≤ 10.5 years of epilepsy and having less frequent seizures (<8) (all *P* < 0.05) (see results in Supplementary Table 1).

## Discussion

We examined the effect of cortico-limbic disruption in the right temporal AH on the ToM deficit; we expected that cognitive-affective disintegration due to cortico-limbic disruption will influence mentalizing about others in a material-specific way (processing of non-verbal versus verbal component of ToM). Although preliminary, present results align with other reports of right-side seizures linked with non-verbal processing deficits. We discuss the following results in serial order: the ToM deficit, specifically the first order ToM deficit linked with the right AH, followed by the results of component-wise analyses that indicated that the right-side seizures show a non-verbal processing deficit (second-order belief of ToM), the right TLE shows spared verbal processing, finally, the result of material specific alteration corroborated the right AH group’s post-surgery decline of non-verbal, belief component of ToM. Additionally, we discuss potential factors contributing to heterogeneity in material-specific post-surgery outcomes.

For the ToM deficit, we observed a significant post-surgery decline in the right AH group's (versus left AH or non-AH) first-order ToM index (pre- and post-surgery first-order belief and metaphor processing). It indicates that right-lateralized cortico-limbic disruption might contribute to first-order ToM deficit (right side localization & right temporal lobe localization did not show this effect, it was observed only for right temporal lobe with AH resection). Change in the second-order ToM (pre- and post-surgery second-order belief and irony processing) did not differ due to any right-side seizure characteristics (seizure side, temporal lobe, or AH resection). Cognitive-affective disintegration right side cortico-limbic disruption (right AH) altered the first-order ToM index (first-order belief and metaphor processing), whereas the second-order ToM probably relied more on the cognitive-verbal processing (second-order belief and irony processing) and therefore was less affected by the right side seizure characteristics. These results align with other reports of visuospatial deficit for right-side impairment,^25,26^ poor non-verbal, somatic-affective processing for right temporal AH ^3,4,6–8^ including studies that support material-specific specialization, that is, non-verbal processing deficit in right AH and verbal deficit in left AH.^27^ We offer explanations based on a functional understanding of the amygdala and hippocampus as crucial parts of the limbic circuitry. Lateralized amygdalar involvement in affective ToM drives our inference of other's emotional states,^28^ and the hippocampus with Tolman-like cognitive map of two-dimensional social space helps navigate social situations requiring ToM.^29^ Therefore, limbic disruption of AH might alter spatial, non-verbal processing, which is critical for the first-order ToM deficit. The non-verbal component in first-order ToM might reflect visuospatial, and somatosensory attribute of physical space useful for mentalizing (e.g. others’ thoughts about an object’s placement in a visuospatial/physical space).

Similarly, metaphor processing as a verbal component of first-order ToM might rely on processing physical somatosensory attributes (e.g. word analogy involving physical characteristics of size, shape, and color). The right side lesion to the amygdala disrupts the region (right posterior superior temporal sulcus) that is involved in using non-verbal cues for mentalizing about others.^30^ Recent studies also indicate a right-hemispheric bias in processing other's visuospatial perspective,^31^ explaining alteration of first-order ToM in the right TL AH rather than left TL AH or non-AH group.

Component-wise analyses enabled further delineation of first and second-order ToM deficit into non-verbal and verbal processing involved in ToM deficit. The non-verbal component (first versus second-order belief) showed a decline in second-order belief processing in the right-side seizure group. These results confirm that as the distance between the perceiver and the person increases from the first (what others think) to the second-order mentalizing (what others think about others), the impoverished somatic-affective inputs will impact the second-order belief processing. The right-side seizure group’s post-surgery decline in second-order belief processing might be due to poor somatic-affective, non-verbal inputs. The verbal component (metaphor versus irony) showed a slight improvement in irony processing in the right TLE group (compared to the non-right TLE group). Compared to metaphors, irony processing (especially sarcastic irony used in the present study) relies predominantly on the left-lateralized semantic network.^32^ When mentalizing about others in semantic terms, the right temporal lobe’s ToM deficit^33^ reflects spared/intact verbal processing compared to the deteriorated non-verbal component of figurative metaphor processing. The summary of component-wise analyses indicates non-verbal deficit (first & second-order belief), specifically in second-order belief ToM for right-side seizures. In contrast, the verbal component (metaphor & irony) specifically, irony processing could be spared or even improved in right TLE.

Post-surgery alteration in ToM in material non-specific processing (conventional ToM assessment) and material-specific processing indicated a need to disentangle distinct components affected in the right side groups (seizure and surgery). The conventionally used combined composite scores amount to a material non-specific processing model (i.e. the non-verbal first-order belief and verbal metaphor compared with the non-verbal second-order belief and verbal irony). The composite scores of material non-specific processing might fail to capture the material-specific deficit of right-side impairment (e.g. post-surgery second-order belief showed a decline, whereas irony processing increased for right-side seizure group). We suspect that producing heterogeneous, mixed results of post-surgery cognitive alterations. As expected, the AH groups showed material-specific alteration (i.e. comparison of the non-verbal component of first and second-order belief with the verbal component of metaphor and irony) that aligned with the material-specific processing model. *Post hoc* analyses corroborated the earlier result; the right AH group showed a post-surgery decline in non-verbal belief processing (first and second-order belief). The ToM deficit in right AH may be due to material-specific processing deficit; impaired non-verbal processing might reduce our ability to mentalize others’ thoughts (especially second-order thoughts, i.e. others’ thoughts about others).

Right-side epilepsy shows material-specific deficit^34^ and right-side TLE shows ToM deficits,^33^ material-specific ToM deficit for right TLE are unclear,^35^ and social deficits in right AH are poorly understood.^36^ Subcortical alteration of right AH is a diagnostic challenge in epilepsy.^8^ The results indicate that cortico-limbic disruption in right AH contributes to first-order ToM deficit that is characteristic of right TLE.^10^ Comparing post-surgery alterations in different ways (component-wise, material non-specific, and material-specific) indicated the usability of the material-specific processing model for understanding post-surgery ToM deficit in right AH. Poor social cognition poses a challenge for identifying structural and functional changes in right TLE,^2,8,37^ possibly due to the hierarchical cortico-limbic circuitry of ToM; identifying material-specific processing deficits breaks down the sub-processes involved in a complex function of ToM. In other words, the delineation of non-verbal and verbal components of the ToM showed the effect of cortico-limbic disruption on the material-specific demands of a complex cognitive function of social cognition and ToM.^38^

In summary, cortical-limbic disruption in right temporal seizure and AH surgery disrupted the cognitive-affective integration required for first-order ToM. The right-lateralized deterioration of non-verbal component might account for the post-surgery decline in first-order ToM (first-order belief and metaphor). In another investigation of post-surgery cognitive-affective disintegration, left-side seizure localization and left unilateral resection showed material-specific alteration (i.e. a noticeable decline in verbal processing compared to non-verbal processing).^39^ The international classification for cognitive disorders in epilepsy calls for studies that offer an internationally applicable understanding of cognitive comorbidities in epilepsy.^40^ Understanding post-surgery cognitive deficits in culturally and linguistically diverse groups is increasingly essential ^41,42^ especially for demystifying cognitive consequences of therapeutic ablation in the form of AH.^43^ Use of culturally sensitive ToM assessment,^4^ the present results suggest that material-specific processing is an essential contributor to cognitive consequences of ToM deficits of the right AH group. Future interventions could improve cognitive-affective integration by addressing the deterioration of non-verbal processing and building on post-surgery verbal processing in ToM function. The present results offer preliminary insights into a cognitive consequence for ToM in right TL AH surgery from an under-represented, developing country with high social embeddedness, warranting higher demand on ToM functions.

These initial results should be considered with limitations; for instance, the order of assessment of first-order ToM and second-order ToM was not counter-balanced; first-order ToM assessment preceded second-order ToM assessment for all participants. This limitation is addressed by delineating the non-verbal component (first-order and second-order false belief) from the verbal component (metaphor and irony). The non-verbal component of second-order belief declined (following first-order belief in first-order ToM assessment) in the right seizure localization group. In contrast, the verbal component of irony processing improved (following metaphor in the first-order ToM) in the right temporal seizure group. The two scores that form one composite index of the ToM component showed a differential effect, indicating that the order effect might not have influenced the results. The current study had 18 participants assessed with average intellectual functions. Participants with borderline intelligence were included because they are considered capable of performing daily living and job-related tasks, show task comprehension and intact speech to verbalize answers and belong to a trainable category. Despite a detailed presurgery neurological evaluation of the participants using inclusion criteria, neuropsychological assessment could not be accessed (e.g. visuospatial memory, language processing). Some have observed that ToM performance is independent of age-related decline in general intelligence.^44^ Although healthy control group was absent, it could have shed light on the ToM assessment in the absence of epilepsy, instead a within-subject comparison where each participant with epilepsy served as their control was used. There was a uniform gap of 4 months given for post-surgery assessment to address practice effects. Lastly, conducted additional analyses addressed sample heterogeneity on factors that could influence material-specific alteration in ToM assessment (see Supplementary material). The cortico-limbic disruptions of selective AH show less cognitive impairment. However, age, sex, education, epilepsy duration, side of seizure, and surgery add heterogeneity to cognitive outcomes, producing mixed results and posing a challenge to identify the processes involved in ToM. It is acknowledge that these results are preliminary, the post-surgery material-specific ToM deficits in the right AH's participants hold true for the right-handed male adults (≥22 years) with average intelligence and school education (middle school) with ≤ 10.5 years of epilepsy and less frequent seizures (>8). There was no a priori sample size estimation; however, the sample size, and the recruitment were comparable with other studies that examined ToM deficits with less than 30 TLE participants.^11,19–21^

Epilepsy is a network disorder that challenges identifying specific structural and functional alterations that contribute to specific post-surgery deficits, especially for complex cognitive functions such as ToM. These preliminary results indicate that non-verbal and verbal components involved in the ToM contribute differentially to post-surgery social deficits associated with right AH. The right AH disruption impairs the non-verbal component of the ToM (e.g. visuospatial cues related to the other person's states). It explains the deterioration of perspective taking in the right temporal AH group. Marr's trilevel framework might be used for extrapolation such as: (i) post-surgery cognitive consequence in a specific function/deficit (i.e. ToM) has a (ii) potential explanation in the form of material-specific processing deficit (non-verbal versus verbal component) attributed to (iii) possible hierarchic neural circuitry implicated in the deficit (i.e. seizure-surgery disruption to right hemispheric side, right temporal lobe, and right temporal with AH). Deciphering the structural and functional changes due to surgical disruption in drug-refractory epilepsy requires neurological, neuropsychological, and neuroimaging inputs. Such interdisciplinary investigation from linguistically diverse populations could improve the understanding of brain structure and cognitive functions and help demystify the heterogeneous nature of cognitive consequences of cortico-limbic resection in TLE.

## Supplementary Material

fcad100_Supplementary_Data

## Abbreviations

AH =: amygdalohippocampectomy
TLE =: temporal lobe epilepsy
ToM =: theory of mind
