# Supplementary material for: Cortico-limbic disruption, material-specificity, and deficits in cognitive-affective theory of mind
Source: Brain Commun. 2023 Apr 24;5(2):fcad100. doi: 10.1093/braincomms/fcad100 (PMC10123397; doi:10.1093/braincomms/fcad100)
Supplement: fcad100_Supplementary_Data [file fcad100_supplementary_data.docx]

**Theory of mind measure**

Culturally suitable and Indian adaptation of measure of theory of mind (ToM) was used (Social Cognition Rating Tools in Indian Setting ^1.^ The assessment included four short stories and two stories on metaphor and irony; each was used to calculate the first-order ToM (FOT) and second-order ToM (SOT) index. The first-order ToM is reflected in first-order ToM or FOT index and consists of two short stories on ToM, and two stories on metaphor, similarly the second-order ToM is reflected in SOT index, which consists of two short ToM stories and two stories on irony. The present analyses excluded ten stories assessing Faux Pas composite index (FPCI), eight audiovisual clips in the vernacular language to compute the social perception index (SPI). The details and instructions are explained in brief.

1. ToM first-order tasks: Sally–Anne task^2^. was adapted to Indian settings. In this story (Shanthi–Ravi task), Ravi and Shanti are playing in a room. When Ravi’s mother calls, he puts the ball in the basket and leaves the room. When he is gone Shanti removes the ball from the basket and places it in her box and leaves. When Ravi returns to the room, he wanted to play with the ball. Participant responds with justification (why) to question about where Ravi will look for the ball, in the box or in the basket. If the subject answers both the question correctly, score will be 1, but if the subject answers the justification question wrong the score will be 0 even if the first question is answered right. Smarties task^3^ was adapted as Sweet box task. In this task the sweet box contains money instead of sweets. The ToM question asked is if a relative or a friend comes in the room and shown the box what will s/he think about the box’s content and why. If the answer to the first question is sweet, the score will be 0 but if the answer is money and justification correct, the score will be 1. Also, if the justification is wrong even after answering the first question correctly the score will be 0.

2. ToM second-order tasks**:** Ice-cream-van task^4^was adapted as Ice-cream man task involving a story of two characters Suresh and Rani. After narrating the story, the participant is asked where does Suresh think Rani has gone to buy the ice cream, and why. If the subject answers correctly (the temple) with justification, the score will be 1, but if he answers temple and cannot justify his answer the score will be 0, also if he answers school the score will be 0. Another widely used task, missing-cookies task^5^ was adapted as Hidden-bananas task. The instructor reads the story involving Asha and her brother Vijay who are sitting in their home at the table and Vijay is eating bananas. When a friend calls Vijay and he goes to meet him outside while Asha keeps the bananas in the cupboard but Vijay sees her doing so from the window. After the story narration, the participant is asked where does Asha think that Vijay would look for the bananas and why? If the participant answers correctly (table) and justifies correctly the score will be 1, but if he answers correctly but cannot justify his answer the score will be 0, also if he answers cupboard the score will be 0.

3. Metaphor-Irony: Metaphor-Irony stories were adapted as Metaphor-Irony stories^6^

**Metaphor - Irony Story no.1 :** The instructor will first read out a story to the subject. Once the story is complete he will ask the subject for any doubts he had regarding the story and then clarify the doubts. If he has understood it well, Instructor will ask him a few questions related to the story which the subject needs to answer.

The instructor will then narrate the story. In the story, Arun leaves his village and goes to a city for higher education. While studying in the city, he puts on a lot of weight, which is very conspicuous. He returns to the village after 6 months of vacation. He goes to see his friend Santosh in his house. Upon seeing him Santosh comments, "**Hey Arun, you have become an elephant!!** At this point, another friend of theirs, Anand comes in. He comments, **"What Arun, didn't anyone feed you in the city?!"**

The instructor will ask for clarification of doubts if any.

Then the instructor will ask a Metaphor question: When Santosh said **" you have become an elephant!”** Did he mean Arun is slim or fat? If the subject answers fat the score is 1 but if he answers slim the score is 0.

Next, the instructor will ask an Irony question: When Anand said "**What Arun, didn't anyone feed you in the city**?!" Did he mean Arun is slim or fat? If the subject answers fat the score is 1 but if he answers slim the score is 0.

**Metaphor - Irony Story no. 2**

The instructor will first read out a story to the subject. Once the story is complete he will ask the subject for any doubts he had regarding the story and then clarify the doubts. If he has understood it well, Instructor will ask him a few questions related to the story which the subject needs to answer.

The instructor will then narrate the story. IN the story Harish, Suraj, and Prashanth are three close friends. One day they decided to have a singing competition. Harish sang a song first, which came out very badly. Suraj commented, "**For a moment I thought a donkey was singing**". Harish asked Prashanth how was his singing, to which he replied, "**Oh, You sang very well with great expertise**"

The instructor will ask for clarification of doubts if any.

When Prashanth said "**Oh, You sang very well with great expertise**" did he mean Harish sang well or not" If the subject asnwers Didn't sing well the score is 1 but if he answers he sang well the score is 0

Next, the instructor will ask a metaphor question: When Suraj said "**For a moment I thought a donkey was singing**" did he mean Harish sang well or not? If the subject asnwers Didn't sing well the score is 1 but if he answers he sang well the score is 0.

**Supplementary Table 1**: Material-specific post-surgery difference in non-verbal (first + second order belief) vs. verbal processing (metaphor + irony) in AH groups examined for key factors (data-split, negative rank).

| Variable | Level | Right AH | Left AH | Non-AH |
| --- | --- | --- | --- | --- |
| Age | Young | n = 2, Z = − 1.34, p = .18 | n = 4, Z = − 1.83, p = .07 | n = 9, Z= − 2.52, **p = .01** |
|  | Old | n = 8, Z = − 2.53, **p = .01** | n = 5, Z= − 2.03, **p = .04** | n = 2, Z= − 1.34, p = .18 |
| Sex | Male | n = 7, Z = − 2.38, **p = .01** | n = 6, Z= − 2.20, **p = .02** | n = 8, Z= − 2.37, **p = .01** |
|  | Female | n= 3, Z = − 1.60, p = .10 | n = 3, Z = − 1.60, p = .10 | n = 3, Z = − 1.60, p = .10 |
| Handedness | Right | n =9, Z= − 2.67, **p = .00** | n = 7, Z= − 2.37, **p = .01** | n = 8, Z= − 2.53, **p = .01** |
|  | Left | n = 1, NA | n = 2, Z = − 1.34, p = .18 | n = 3, Z = − 1.34, p = .18 |
| Intelligence | Low | n = 0, NA | n = 5, z= − 2.03, **p = .04** | n = 7, z= − 2.20, **p = .02** |
|  | Midlevel | n = 10, Z = − 2.81, **p = .00** | n = 4, Z = − 1.82, p = .06 | n = 4, Z = − 1.82, p = .06 |
| Education | ≤ 8^th^ | n = 3, Z = − 1.60, p = .10 | n = 6, Z= − 2.20, **p = .02** | n = 6, Z= − 2.03, **p = .04** |
|  | Higher | n = 7, Z = − 2.37, **p = .01** | n = 3, Z = − 1.60, p = .10 | n = 5, Z= − 2.03, **p = .04** |
| Duration | ≤ 10.5 y | n = 7, Z = − 2.37, **p = .01** | n = 2, Z = − 1.34, p = .18 | n = 6, Z= − 2.20, **p = .02** |
|  | Higher | n = 3, Z = − 1.60, p = .10 | n = 7, Z= − 2.37, **p = .01** | n = 5, Z = − 1.82, p = .06 |
| Seizure freq. | Low | n = 8, Z = − 2.52, **p = .01** | n = 5, Z= − 2.03, **p = .04** | n = 3, Z = − 1.63, p = .10 |
|  | High | n = 2, Z = − 1.34, p = .18 | n = 4, Z = − 1.82, p = .06 | n = 8, Z= − 2.37, **p = .01** |

1. Mehta UM, Thirthalli J, Naveen Kumar C, et al. Validation of Social Cognition Rating Tools in Indian Setting (SOCRATIS): A new test-battery to assess social cognition. *Asian J Psychiatr*. 2011;4(3):203-209.
2. Wimmer H, Perner J. Beliefs about beliefs: representation and constraining function of wrong beliefs in young children's understanding of deception. *Cognition.* 1983;13(1):103-128.
3. Perner J, Wimmer H. “John thinks that Mary thinks that…” attribution of second-order beliefs by 5- to 10-year-old children. *Journal of Experimental Child Psychology*. 1985;39(3):437-471.
4. Perner J, Leekam SR, Wimmer H. Three-year-olds’ difficulty with false belief: The case for a conceptual deficit. *British Journal of Developmental Psychology*. 1987;5:125-137.
5. Stone VE, Baron-Cohen S, Knight RT. Frontal lobe contributions to theory of mind. J Cogn Neurosci. 1998;10(5):640-656.
6. Drury VM, Robinson EJ, Birchwood M. 'Theory of mind' skills during an acute episode of psychosis and following recovery. *Psychol Med.* 1998;28(5):1101-1112.
